# Prevalence of Intestinal Parasites and *Salmonella typhi* among Food Handlers Working in Catering Establishments of Public Institutes Found in Dawuro Zone, South-Western Ethiopia

**DOI:** 10.1155/2021/8889302

**Published:** 2021-01-13

**Authors:** Abera Kumalo, Eyasu Gambura, Terfe Dodicho, Khawaja Shakeel Ahmed, Tamrat Balcha, Bahailu Beshir, Misrak Abraham

**Affiliations:** ^1^Department of Medical Laboratory Science, College of Health Sciences and Medicine, Wolaita Sodo University, Sodo, Ethiopia; ^2^Department of Environmental Health under School of Public Health, College of Health Sciences and Medicine, Wolaita Sodo University, Ethiopia; ^3^School of Public Health, Wolaita Sodo University, Dawuro Tarcha Campus, Ethiopia; ^4^Department of Pharmacy, College of Health Sciences and Medicine, Wolaita Sodo University, Ethiopia; ^5^Department of Psychology, School of Educational & Behavioral Sciences, Wolaita Sodo University, Sodo, Ethiopia; ^6^Wolaita Zone Livestock and Fishery Resource Department, Sodo, Ethiopia

## Abstract

**Background:**

Food borne disease, which is the result of ingestion of foodstuffs contaminated with microorganisms, parasites, or chemicals, encompasses a wide spectrum of illness and public health problem worldwide. Ethiopia is placed on second, third, and fourth position due to the highest burden of ascariasis, hookworm, and trichuriasis, respectively, in sub-Saharan Africa. The present study is aimed at determining the prevalence of *Salmonella typhi* and intestinal parasites among food handlers working in catering establishments of public institutes found in Dawuro Zone, South-Western Ethiopia.

**Methods:**

A cross-sectional study is conducted among food handlers working in catering establishments of public institutions found in Dawuro Zone from March to July 2019. The data was collected by using pretested structured questionnaires. Stool and blood samples were taken from each participant for parasitic examinations concurrently using direct and modified formol ether concentration wet smear techniques and Widal test by slide test and tube serial dilution technique, respectively. The data entered into Epi info version 3.5.1 and then exported into SPSS window version 20.0 for analysis. Logistic regressions were performed to assess the association between binary outcomes and different explanatory variables. *P* value <0.05 was considered statistically significant.

**Result:**

The study included 402 (293 females and 109 males) food handlers. Of 402 stool specimens, 20.4% were found to be positive for different parasite species, comprising protozoa (35.9%) and helminths (64.1%). *A*. *lumbricoides* was the most prevalent parasite (8.0%), followed by *E*. *histolytica/dispar* (4.23%). Twenty-six (6.5%) of food handlers were positive for Widal test. Ages ≥ 40 years (AOR: 0.436; 95% CI: 0.203, 0.937), attending no education (AOR: 2.142; 95% CI: 1.048, 4.378), not washing hands after using latrine (AOR: 4.355; 95% CI: 1.771, 10.708), not covering mouth with tissue paper (AOR: 0.530; 95% CI: 0.312, 0.899), no medical checkup (in the last four months) (AOR: 0.278; 95% CI: 0.116, 0.667), and untrimmed fingernails (AOR: 0.382; 95% CI: 0.229, 0.635) were significantly associated with intestinal parasitic or *Salmonella typhi* infections.

**Conclusion:**

The prevalence of intestinal parasitic infection (IPI) and *Salmonella* among food handlers in the present study is relatively high compared to other different studies conducted in developed and developing countries. Therefore, biannually screening of food handlers for intestinal parasites (IPs) and periodic deworming of infected cases along with provision of food safety measures focusing on personal hygiene and environmental sanitation are recommended to control the parasitic infection in food handlers.

## 1. Introduction

A food borne disease, which is the result of ingestion of foodstuffs contaminated with microorganisms or chemicals, encompasses a wide spectrum of illnesses and a growing public health problem worldwide (1–3). According to the Ministry of Health (MOH) of Ethiopia, food borne disease implies food that is not handled in clean conditions; hence, the food becomes contaminated by disease causing microorganisms. These microorganisms grow and multiply in the food; hence, people who consume this food may get poisoned or infected by organisms in the food (4). Thus, an estimated 70% of cases of diarrheal diseases are associated with the consumption of contaminated food. Diarrheal diseases are mostly caused by food or waterborne microbial pathogens and remain the leading causes of illness and deaths, with an estimated killing of 1.9 million people annually worldwide. Yet, it is expected that a large number of illnesses remain underreported because only the most serious cases are usually investigated (3, 5).

In worldwide, about one-third of the total population is projected to be infected with intestinal parasites, from which the leading number of people are living in tropical and subtropical parts of the world (6, 7). Centers for Disease Control and Prevention (CDC) estimated that each year, 48 million people get sick, 128,000 are hospitalized, and 3000 die from a foodborne disease (8, 9). About 819 million people are infected with *Ascaris lumbricoides* (*A*. *lumbricoides*), 464.6 million people with *Trichuris trichiura* (*T*. *trichiura*), 438.9 million people with hookworm infection (7, 10), 500 million people with *Entamoeba histolytica* (*E. histolytica*), and 2.8 million people are infected with *Giardia lamblia* (*G*. *lamblia*) (7, 11).

In USA, food borne diseases cause an estimated 76 million illnesses, 325,000 hospitalizations, and 5,000 deaths each year. Experts estimate that the yearly cost of all food borne diseases in USA is $5 to $6 billion in direct medical expenses and lost productivity. *Salmonella* infections alone in USA account for $1 billion yearly in direct and indirect medical costs. Nevertheless, in most developing countries, reliable statistics on food borne diseases are not available due to poor or nonexistent reporting systems (3).

In Ethiopia, the case of intestinal parasitosis is highly common, like that of other developing countries which are found in sub-Saharan Africa. It is assumed that one-third of Ethiopians are infected with *A*. *lumbricoides*, one quarter is infected with *T*. *trichiura*, and one in eight lives with *hookworm*. Due to this, Ethiopia is placed in second, third, and fourth position with the highest burden of ascariasis, hookworm, and trichuriasis, respectively, in sub-Saharan Africa (7, 12). According to the different studies, the most prevalent intestinal protozoan parasites in Ethiopia are *Giardia lamblia* and *Entamoeba histolytica/dispar*. Helminthic infection includes *Ascaris lumbricoides*, *Trichuris trichuria*, and *Taenia saginata* (9, 13–15). In addition to intestinal parasitosis, *Salmonella typhi* is also one of the leading causes of food and water borne gastroenteritis in human. Globally, it is one of the important public health concerns with estimated 16 million new cases and 600,000 typhoid fever deaths each year (16, 17).

A food handler is a person with any job that requires him/her to handle unpackaged foods or beverages and involved in preparing, manufacturing, serving, inspecting, or even packaging of food and beverage items and also those who come in contact with eating or cooking utensils or other equipment used in the handling, preparation, service, or sale of food (3, 18). All food handlers are required to use proper hygiene and sanitation methods when working with food (4). According to the world health status review, the health problem of developing nations is primarily interrelated to sanitation issue. Thus, various factors such as the general sanitary standards of the house, the proper use of sanitary facilities like latrines and hand washing lavatories, refuse management systems, and dishwashing facilities affect food safety in food establishments (19–21). Food handling, preparation, and service practices are other important factors in determining the safety of food. Conditions of cooking utensils and food storage systems (time and temperature) as well as food handlers' knowledge and practices similarly affect food safety directly or indirectly (4, 15, 22–25). The fact that less evidence is obtained in the literature in the current study area regarding the intestinal parasites and *Salmonella* prevalence as well as associated factors, so the current study is aimed at assessing the prevalence of *Salmonella* and intestinal parasite among food handlers who are serving in catering establishment of public institutes found in Dawuro Zone, South-Western Ethiopia.

## 2. Methods

### 2.1. Study Area, Design, and Period

A cross-sectional study design was conducted from March to July 2019 in three towns found in Dawuro Zone of South-Western Ethiopia, namely, Tarcha, Waka, and Gessa towns. Tarcha is the administrative center of Dawuro Zone which is 476 km away from the capital city, Addis Ababa. Waka and Gessa towns are the district centers of Maraka and Loma districts, Dawuro Zone, which are found to be 460 km and 445 km away from the capital city, Addis Ababa, respectively, in road Sodo to Tarcha.

### 2.2. Study Population

Sampled food handlers working in catering establishments of public institutions found in Tarcha, Waka, and Gessa towns, Dawuro Zone of South-Western Ethiopia are involved in this study.

### 2.3. Inclusion Criteria and Exclusion Criteria

Food handlers working in selected food establishment, who have direct contact with food and drinks and who have given an informed consent, were included in the study. Those who had taken antiparasitic drugs and antibiotic within three weeks prior to the study were excluded.

### 2.4. Sample Size Determination

Sample size that was determined using the formula for single population proportion using the prevalence of intestinal parasite among food handlers was 44.1% (26) from Yebu with a 95% confidence interval, a margin of error of 5%. An initial sample size was 379. By adding 10% nonresponse rate, the final sample size will be 417.

### 2.5. Sampling Technique

**Figure 1:**
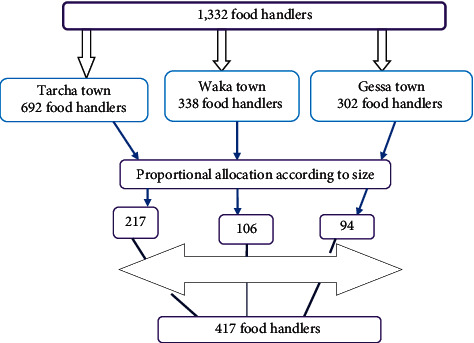
Flow chart of sampling techniques.

The study participant was 417 food handlers who fulfilled the inclusion criteria that currently working in the food catering establishment of public institutions in Tarcha, Waka, and Gessa towns. Prior to the study, survey study was conducted to know the number and types of the public institution found in each town. Also, the total number of food handlers working in each catering establishment of the public institutes in the respective towns was identified. Then, the total sample size was divided proportionally for the three towns depending on the total number of the food handlers of the public institutes found in each town. Finally, 417 food handlers were included in the study by systematic random sampling until the total sample size met (Figure 1).

### 2.6. Data and Specimen Collection

The data was collected through face to face interview using pretested structured questionnaires. The structured interview questionnaire was taken 15 minutes with closed-ended questions and predetermined response options which are developed specifically for this study. The questionnaire contains sociodemographic status, knowledge of food handling, practice of food handling, and personal hygiene. The questionnaire was adopted from the World Health Organization (WHO) food safety checklist and other related literatures (9, 20, 23, 27). After interviewing, respondents were asked to collect a small amount of fresh stool sample (2 g or 4 ml if diarrhea) with a clean and tight-lid sample container after orienting on how to collect the stool specimen by attending laboratory technologist. All specimens were immediately transported using cold box to the Microbiology and Parasitology Laboratory of Wolaita Sodo University for analysis.

### 2.7. Specimen Handling and Processing

Each stool sample was smeared and tested in doubled manner. Parasitological microscopic stool examination was immediately performed by emulsifying uniformly about a matchstick size of stool (2 mg) in a drop of normal saline (0.85% NaCl) on one end of a glass slide, and an iodine wet mount was mainly used to stain the nucleus of the protozoa cysts in the other end of the same slide. Sedimentation technique was performed using the readily modified formalin-ether sedimentation technique. In brief, 1 g of stool was placed in 15 ml conical tube containing 7 ml of 10% formol water, gently emulsified and sieved using 65 mm plastic strainer. The sieved sample was transferred into another conical tube containing 9 ml of 10% formol water and 3 ml of ethyl acetate and centrifuged at 3000 rpm. The supernatant was decanted by inverting the tube, and the last drop allowed to sediment by gravity for 15 min. The sediment was then poured on slide, covered with cover glass (22 mm × 22 mm), and examined microscopically under low (10x) with the condenser iris closed sufficiently to give good contrast and high (40x) objective lenses. Eggs and larvae of helminths and cysts and trophozoites of protozoan were assessed by propagule size, shape, cell wall width, and distinctive internal characteristics (9, 28).

### 2.8. Widal Test

Two milliliters of venous blood was collected by the data collector from food handlers for Widal test. Serum is prepared; allow the whole blood to clot at room temperature for at least one hour or centrifuge the clotted blood for 10 minutes at 2000 rpm. The Widal test was done using *S*. *typhi* O and H antigens according to the manufacturer's instruction. In brief, the test was done by mixing one drop of serum with one drop of O and H antigens separately on glass slide. After rocking the slide back and forth, the mixture was observed for macroscopic agglutination. In addition to slide (screening) test, serial (tube) dilution tests done by using patient serum are made in physiologic saline. An antigen suspension is added to the mixture and incubated for a certain period of time. Finally, the highest dilution giving agglutination reaction (titer) is determined. Widal test is reported by giving the titer for both O and H antibodies. Positive result was reported by the presence of agglutination within one minute, and negative result was reported by the no agglutination (28, 29).

### 2.9. Methods of Data Processing and Analysis

The coded data was checked, cleaned, and entered into the epidemiological information software (Epi info version 3.5.1) to ensure data accuracy and then exported into SPSS window version 20.0 (SPSS Inc., Chicago, IL) for analysis. Descriptive statistics were manipulated for all variables. Bivariate analysis was first conducted for each potentially explanatory risk factor to see associations to select for multivariate analysis. Variables that had a *P* value <0.25 in bivariate analysis were run in multivariable logistic regression. Multiple logistic regressions were performed to assess the association between binary outcomes and different explanatory variables. The strength of association was interpreted using odds ratio and confidence interval. *P* value <0.05 was considered statistically significant in this study.

### 2.10. Data Quality Control

Questionnaire was prepared in English and translated to local language (Amharic and Dawuro languages) and retranslated back to English. Data collectors were trained ahead of the actual data collection period. The training was focused on familiarizing interviewers with the questionnaire and giving them the opportunity to practice using it. Well-experienced and trained laboratory technologist was recruited for laboratory examination. Before the actual stool specimen's examination for the study subjects, pretest was conducted on 5% of stool samples collected from patients attending Tarcha health centers to look the reliability/reproducibility of stool examination procedure by the laboratory technologist with two different microscopes. In addition to slide (screening) test, serial (tube) dilution tests done by using patient serum are made in physiologic saline. An antigen suspension is added to the mixture and incubated for a certain period of time. Finally, the highest dilution giving agglutination reaction (titer) is determined. Widal test is reported by giving the titer for both O and H antibodies.

At time of data collection filled questionnaires was checked for completeness, accuracy, and consistency of information by the supervisor on daily basis, and typing errors were manually edited. Any ambiguity and other problems of data collectors were addressed by communicating with the data collectors before the following week.

## 3. Results

### 3.1. Study Population Characteristics

The age and sex distribution of food handlers are presented in Table 1. The study included 402 (293 females and 109 males) food handlers with a response rate of 96.4%. Majority of the food handlers (61.2%) were found in the age group of 20–40 years and had a primary educational level of around 43.8%. Job position in the catering establishment indicates almost more than half had has cooking (60.2%) position, and waiter position indicates 16.4%. The majority (57.5%) of food handlers had served for 1-2 years.

### 3.2. Etiologic Agents

#### 3.2.1. Intestinal Parasites

Direct microscopic and concentration techniques were used for identifying intestinal parasites from the 402 stool specimens. Of 402 stool specimens, 20.4% were found to be positive for different parasite species, comprising protozoa (35.9%) and helminths (64.1%). *A*. *lumbricoides* was the most prevalent parasite (7.9%), followed by *E*. *histolytica/dispar* (4.23%) and then *G*. *lamblia* (2.74%). Less frequent identified intestinal parasite spp. was *S*. *stercoralis and Taenia species*; each accounted for 0.49% and 0.75% of the total isolates. Mixed intestinal parasites (*A*. *lumbricoides*, *G*. *lamblia*, *and Tania species*) were detected in 5 (1.2%) of the participants (Table 2).

#### 3.2.2. Salmonella

Out of 402 food handlers screened, Widal test result showed that 26 (6.5%) food handlers were positive for O or/and H antigens of *Salmonella typhi*.

### 3.3. Hygienic Practice of Food Handlers

The hygienic practice and knowledge of food handlers are presented in Table 3. In personal hygiene-related practices, more than half of the participants responded keeping ready to eat foods in clean container and covering it properly (348, 86.6%), checking ingredients' expiry date before using for food preparation (386. 96%), covering mouth with tissue paper, when coughing or sneezing accidentally during food preparation (280, 69.7%), and storing foods which are capable of supporting microorganisms' growth in the refrigerator (270, 67.2%). However, certified in food preparation and handling is low which accounts 99 (24.6%). In the other part, 250 (62.2%), 291 (72.4%), and 290 (72.1%) food handlers had a habit of hand washing with water only after visiting the latrine, touching dirty materials, and touching body parts, respectively. A few number (19.2% and 25.6%) of food handlers had a habit of medical checkup and habit of covering hair during food preparation, but they are good at shoe wearing habit (294, 73.1%) and trimmed fingernail status (248, 61.7%).

### 3.4. Knowledge of Food Handlers

With regard to knowledge-related factors, food handlers (74.9%) had little knowledge to use of potable water that decreases the transmission of intestinal parasites/*Salmonella typhi.* Among food handlers who believed that proper use of latrine (33.1%), cleaning of utensils (37%), cleaning of the kitchen floor (21.6%), proper clothing (34%), and food hygiene and safety training (27%) had a good knowledge that decreases the transmission of intestinal parasites/*Salmonella typhi* (Table 4).

### 3.5. Factors Associated with Intestinal Parasitic and *Salmonella* Infections

As shown in Table 5, different factors were assessed for possible association with intestinal parasitic and *Salmonella typhi* infection among the study participants. Among the total variables included in the bivariate logistic analysis, eight variables (age (in years), level of education, hand washing practice after visiting the latrine, cover mouth with tissue paper, when coughing or sneezing accidentally during food preparation, store foods which are capable of supporting microorganisms growth in the refrigerator, medical checkup (in the last four months), and fingernail status were associated with IPIs at a *P* value <0.25. All those variables that were associated with IPIs and *Salmonella typhi* at a *P* value <0.25 in bivariate logistic regression analysis were computed in a multivariate logistic regression analysis. The results of multivariable logistic analysis showed that ages ≥ 40 years (AOR: 0.436; 95% CI: 0.203, 0.937), attending no education (AOR: 2.142; 95% CI: 1.048, 4.378), not washing hands after visiting the latrine (AOR: 4.355; 95% CI: 1.771, 10.708), not covering mouth with tissue paper, when coughing or sneezing accidentally during food preparation (AOR: 0.530; 95% CI: 0.312, 0.899), not storing foods which are capable of supporting microorganisms' growth in the refrigerator (AOR: 0.465; 95% CI: 0.275, 0.787), without medical checkup (in the last four months) (AOR: 0.278; 95% CI: 0.116, 0.667), and untrimmed fingernails (AOR: 0.382; 95% CI: 0.229, 0.635) were significantly associated with intestinal parasites or *Salmonella typhi* infections (*P* value <0.05) (Table 5).

## 4. Discussion

The spread of disease via food handlers is a public and persistent problem globally. Infection of asymptomatic persons, especially workers dealing with food (food handlers), could become a potential cause of dissemination of variety of pathogens including intestinal parasites and *Salmonella* (9, 29, 30). Intestinal parasitic infection is one of the problems that affect human health, especially in developing countries. If food handlers are contaminated with parasites which have the potential to be directly transmitted from one person to another, they can transmit contamination to food, dishes, and finally to the people who use them (30). Different studies have been conducted in the field of intestinal parasite prevalence in food handlers. However, published information about food borne pathogens among food handlers in our study area is scarce. Therefore, this study was undertaken to determine the prevalence of *Salmonella* and intestinal parasites among food handlers working in catering establishments of public institutes found in Dawuro Zone of South-West Ethiopia.

The prevalence of IPIs in the present study was 20.4%. The findings of our study were consistent with the studies done in Haramaya University cafeterias, Eastern Ethiopia, Gondar town, North West Ethiopia, Madda Walabu University, Aksum town, Northern Ethiopia, and Accra, Ghana, with a prevalence of 25.2% (9), 25% (31), 25.3% (32), 14.5% (33), and 15% (34), respectively. On the other hand, the prevalence found in our study is low compared to the previous studies conducted elsewhere in Ethiopia and other parts of the world, like Wolaita Sodo town, Yebu town, Jimma zone, Bahir Dar town, Addis Ababa, Gondar, Abeokuta, Nigeria, and Mekele which reported prevalence of 33.68% (30), 44.1% (26), 41.1% (14), 45.3% (35), 29.1% (36), 97% (37), and 52.4% (25), respectively. The studies done in Sai-Yok, Thailand, and North India reported a prevalence of 10.3% (38) and 1.3 to 7% (39), respectively, which is low compared to our study. Reporting of such variations is large due to the difference in sociodemographic characteristics, geographical location, safe water supply, ignorance of health-promotion practices, food hygiene, and safety training.

In this study, the most frequently isolated intestinal parasite was *A*. *lumbricoides* (8.0%) from helminthes and *E*. *histolytica/dispar* (4.2%) was the most predominant from protozoan parasites. The findings of our study are in line with other studies conducted in different parts of Ethiopia, like Wolaita Sodo (6.25% and 4.51%) (30), Hawassa (9.6% and 2.2%), (29), and in Gondar (18.11% and 1.6%) (36). But, it was in disagreement with a study reported from Yebu, southwestern Ethiopia, in which *A*. *lumbricoides* (17.8%) was the leading parasite from helminths and *G. intestinalis* (5.9%) predominant from protozoan (26). The study conducted in Mekele, Ethiopia, at different time periods, reported results of 32.3% (40) and 36.6% (15) for *A*. *lumbricoides* and *E*. *histolytica/dispar*, respectively, which is different from our study, while the study done in Bahir Dar town of Ethiopia, *E*. *histolytica/dispar* with 12.76 (14) was predominant compared to *A*. *lumbricoides*. The difference in the frequency and type of parasites may be due to differences in geographical location and environmental conditions.

This study also tried to identify S. *typhi* infection from serum sample of food handlers by using Widal test for “O” or/and “H” antigens of *Salmonella typhi*. Overall, 26 (6.5%) food handlers were found reactive with *Salmonella typhi* by “O” or/and “H” antigens. This result is in close agreement with the study conducted among food handlers at the University of Hawassa (8.1%) (29). However, in a study conducted in Bahir Dar, (1.6%) food handlers were found infected with *S*. *typhi* (14); in Motta town of North West Ethiopia, the percentage of infection was 2.5% (41), and in Addis Ababa, it was 3.5% (35). The study done by Andargie et al. in Gondar town reported no presence of *Salmonella* species in food handlers (36). The variation observed could be due to the differences in the socioeconomic conditions, type of diagnostic sensitivity, epidemiological difference, seasonal variation, and the differences in hygiene of the individuals and the working environment.

As shown in Table 5, different factors were assessed for possible association with intestinal parasitic and *Salmonella typhi* infection among the study participants. The odds ratio of being positive for IPIs or *Salmonella typhi* infections was 0.436 times higher among food handlers who had age greater than 40 years than those who had age less than 20 years (*P* = 0.034; AOR: 0.436 95% CI: 0.203, 0.937). Food handlers who had no education were 2.142 times more likely to have increased intestinal parasitic and *Salmonella typhi* infection as compared to respondents who had secondary and above education (AOR: 2.142; 95% CI: 1.048, 4.378). The findings of our study are supported by earlier studies conducted elsewhere in Ethiopia (9, 31, 33). This may be suggested by due to lack of education among food handlers that makes them more vulnerable to parasitic and enteric infection, as they are short of knowledge about food safety and transmission of parasitic and enteric infection.

The odds ratio of being positive for IPIs or *Salmonella typhi* infections is 4.355 times higher among food handlers who did not wash their hands after using latrine, compared to those who washed their hands with water and soap (*P* = 0.001; AOR: 4.355; 95% CI: 1.771, 10.708). This finding is in line with earlier studies conducted elsewhere in Ethiopia (9, 23). This may be due to lack of sanitary materials, level of education, and lack of personal hygiene training.

Not covering mouth with tissue paper during coughing or sneezing accidentally as well as during food preparation and not storing food which are capable of supporting microorganisms' growth in the refrigerator were identified as significant associated risk factors among food handlers infected by IPs or *Salmonella typhi*. In this study, the odds ratio of being positive for IPs or *Salmonella typhi* infections is 0.278 times higher among those food handlers who did not had medical checkup (in the last four months) compared to those who had (*P* = 0.004; AOR: 0.278; 95% CI: 0.116, 0.667). Earlier studies also suggest that medical checkup of food handlers must be done in every 3, 6, and 9 months (9). Currently, the Ministry of Health of Ethiopia also encourages food handlers to renew their medical certificate in every 3 months (33). The odds ratio of IPs or *Salmonella typhi* infections is 0.382 times higher among food handlers who had untrimmed fingernails compared to those who had trimmed fingernails (*P* = 0.000; AOR: 0.382; 95% CI: 0.229, 0.635). Other similar studies also support our findings; untrimmed fingernails can be a risk factor for IPIs among food handlers (9, 14, 33). Untrimmed fingernails could serve as a vehicle for transport of intestinal parasite or enteric bacteria from source to food due to difficulty of cleaning (9, 20). But the present study did not attempt to assess the parasite carriage of the fingernail contents.

In our study, there were some limitations.

1. Due to lack of antigen or molecular tests, *Entamoeba histolytica* and *Entamoeba dispar* were not separated
2. The fingernail contents were not investigated for ova/cyst of parasites
3. A specific parasitic diagnostic method was not employed for identification of specific parasites, like string test for *Giardia lamblia* and adhesive scotch tape for *E*. *vermicular*
4. The gold standard test for identification of *Salmonella* is culture; however, due to lack of laboratory setup in study area, we conducted only screening test of *Salmonella* by Widal test

## 5. Conclusion

In conclusion, the prevalence of IPIs among food handlers in our study was 82 (20.4%), which is relatively high in comparison to other different studies conducted in developed and developing countries. *A. lumbricoides* was the most prevalent parasite (7.9%), followed by *E*. *histolytica/dispar* (4.23%) and *G*. *lamblia* (2.74%). The study also identified *Salmonella typhi infection.* The prevalence of *Salmonella typhi* by Widal test was 26 (6.5%). Since most of the intestinal parasites are transmitted through feco-oral route, food handlers can be an important source of infection to the general population. Among the variables included in the bivariate logistic analysis, eight different variables like (age (in years) level of education, hand washing practice after using latrine, covering mouth with tissue paper when coughing or sneezing accidentally during food preparation, stored food which are capable of supporting microorganisms' growth in the refrigerator, medical checkup (in the last four months), and fingernail status were identified as significant risk factors which are making food handlers susceptible to intestinal parasites in our study area. Therefore, biannually screening of food handlers for intestinal parasites (IPs) and periodic deworming of infected cases along with provision of food safety measures focusing on personal hygiene, environmental sanitation, and periodical medical checkups are recommended to control the parasitic infection in food handlers.

## Figures and Tables

**Table 1:** The sociodemographic characteristics of food handlers working in catering establishments in Dawuro Zone, South-Western Ethiopia, March to July 2019 (*N* = 402).

Variables	Response category	Frequency	Percent (%)
Sex	Male	109	27.1
	Female	293	72.9
Age (in years)	<20	98	24.4
	20-40	246	61.2
	≥40	58	14.4
Level of education	No education	61	15.2
	Primary	176	43.8
	Secondary and above	165	41.0
Job position in the catering establishment	Cooking	242	60.2
	Cleaning utensils	94	23.4
	Waiter	66	16.4
Year of service	<1 year	107	26.6
	1-2 years	231	57.5
	≥2 years	64	15.9
Monthly income in Birr	<1100 ETB	264	65.7
	≥1100 ETB	138	34.3

**Table 2:** Prevalence of intestinal parasites and Salmonella of food handlers working in catering establishments in Dawuro Zone, South-Western Ethiopia, March to July 2019 (*N* = 402) (*N* = 82 and *N* = 26 Salmonella).

Parasites identified	Frequency	Percent (%)
*A*. *lumbricoides*	32	8.0
*S*. *stercoralis*	2	0.49
*Giardia lamblia*	11	2.74
*T*. *trichuria*	5	1.2
*Hookworm species*	7	1.74
*E. histolytica/dispar*	17	4.23
*Taenia species*	3	0.75
Mixed infections	5	1.2
Total	82	20.4
*Salmonella typhi* (*Widal test*)	26	6.5

**Table 3:** Personal hygiene-related factors of food handlers working in catering establishments in Dawuro Zone, South-Western Ethiopia, March to July 2019 (*N* = 402).

Variables	Response category	Frequency	Percent (%)
Keep ready to eat foods in clean container and cover it properly	Yes	348	86.6
	No	54	13.4
Check ingredients' expiry date before using for food preparation	Yes	386	96
	No	16	4
Cover mouth with tissue paper, when coughing or sneezing accidentally during food preparation	Yes	280	69.7
	No	122	30.3
Store foods which are capable of supporting microorganisms' growth in the refrigerator	Yes	270	67.2
	No	132	32.8
Certified in food preparation and handling	Yes	99	24.6
	No	303	75.4
Hand washing practice after visiting the latrine	Not wash	90	22.4
	Water only	250	62.2
	Water and soap	62	15.4
Hand washing practice after touching dirty materials	Not wash	83	20.6
	Water only	291	72.4
	Water and soap	28	7.0
Hand washing practice after touching body parts	Not wash	90	22.4
	Water only	290	72.1
	Water and soap	22	5.5
Habit of covering hair during food preparation	Yes	103	25.6
	No	299	74.4
Medical checkup	Yes	77	19.2
	No	325	80.8
Fingernail status	Trimmed	248	61.7
	Untrimmed	154	38.3
Shoe wearing habit	Yes	294	73.1
	No	108	26.9

**Table 4:** Knowledge-related factors of food handlers working in catering establishments in Dawuro Zone, South-Western Ethiopia, March to July 2019 (*N* = 402).

Variables	Response category	Frequency	Percent (%)
Use of potable water decreases the transmission of intestinal parasites/*Salmonella typhi*	Yes	101	25.1
	No	301	74.9
Proper use of latrine decreases the transmission of intestinal parasites/*Salmonella typhi*	Yes	133	33.1
	No	269	66.9
Cleaning of utensils decreases the transmission of intestinal parasites/*Salmonella typhi*	Yes	149	37
	No	253	63
Cleaning of the kitchen floor decreases the transmission of intestinal parasites/*Salmonella typhi*	Yes	87	21.6
	No	315	78.4
Food hygiene and safety training decrease the transmission of intestinal parasites/*Salmonella typhi*	Yes	109	27
	No	293	73
Proper clothing decreases the transmission of intestinal parasites or *Salmonella typhi*	Yes	137	34
	No	265	66

**Table 5:** Multivariable logistic regression on associated risk factors with intestinal parasites and *Salmonella typhi* infection among food handlers working in catering establishments in Dawuro Zone, South-Western Ethiopia, March to July 2019 (*N* = 402).

Variables	Response category	Positive for intestinal parasites and *Salmonella*		*P* value	AOR (95% CI)
		Yes	No		
Age (in years)	<20	18	80		1
	20-40	55	191	0.239	0.602 (0.258-1.402)
	≥40	35	23	0.034	0.436 (0.203-0.937)
Level of education	No education	36	25	0.037	2.142 (1.048-4.378)
	Primary	47	129	0.768	1.090 (0.614-1.936)
	Secondary and above	25	140		1
Hand washing practice after visiting the latrine	Not wash	59	31	0.001	4.355 (1.771-10.708)
	Water only	42	208	0.131	2.199 (0.790-6.121)
	Water and soap	7	55		1
Cover mouth with tissue paper, when coughing or sneezing accidentally during food preparation	Yes	44	238		1
	No	64	58	0.018	0.530 (0.312-0.899)
Store foods which are capable of supporting microorganisms' growth in the refrigerator	Yes	27	243		1
	No	81	51	0.004	0.465 (0.275-0.787)
Medical checkup (in the last four months)	Yes	15	62		1
	No	93	232	0.004	0.278 (0.116-0.667)
Fingernail status	Trimmed	30	218		1
	Untrimmed	78	76	0.000	0.382 (0.229-0.635)

## Data Availability

All data generated or analyzed during this study are included in this manuscript.

## Abbreviations

AOR: Adjusted odds ratio
CL: Confidence level
IPIs: Intestinal parasitic infections
WHO: World Health Organization.
